# Spatio-temporal spread and evolution of influenza A (H7N9) viruses

**DOI:** 10.3389/fmicb.2022.1002522

**Published:** 2022-09-15

**Authors:** Zhibin Shi, Lili Wei, Pengfei Wang, Shida Wang, Zaisi Liu, Yongping Jiang, Jingfei Wang

**Affiliations:** State Key Laboratory of Veterinary Biotechnology and National Data Center of Animal Infectious Diseases, Harbin Veterinary Research Institute, Chinese Academy of Agricultural Sciences, Harbin, China

**Keywords:** influenza virus, H7N9, spatio-temporal pattern, spread, evolution, risk

## Abstract

The influenza A (H7N9) virus has been seriously concerned for its potential to cause an influenza pandemic. To understand the spread and evolution process of the virus, a spatial and temporal Bayesian evolutionary analysis was conducted on 2,052 H7N9 viruses isolated during 2013 and 2018. It revealed that the H7N9 virus was probably emerged in a border area of Anhui Province in August 2012, approximately 6 months earlier than the first human case reported. Two major epicenters had been developed in the Yangtze River Delta and Peral River Delta regions by the end of 2013, and from where the viruses have also spread to other regions at an average speed of 6.57 km/d. At least 24 genotypes showing have been developed and each of them showed a distinct spatio-temporal distribution pattern. Furthermore, A random forest algorithm-based model has been developed to predict the occurrence risk of H7N9 virus. The model has a high overall forecasting precision (> 97%) and the monthly H7N9 occurrence risk for each county of China was predicted. These findings provide new insights for a comprehensive understanding of the origin, evolution, and occurrence risk of H7N9 virus. Moreover, our study also lays a theoretical basis for conducting risk-based surveillance and prevention of the disease.

## Introduction

In February 2013, human infections with a novel avian influenza A (H7N9) virus were reported in China (Gao et al., 2013). Since then, five epidemic waves of human infections with the H7N9 virus have occurred in China, resulting in 1,565 laboratory confirmed cases and 615 fatal cases, with a case fatality rate of ∼40% (Su et al., 2017; Artois et al., 2018; Xiang et al., 2018; Zhu et al., 2018). Therefore, a great concern about a novel influenza pandemic has been raised by this virus. Initial investigations on the human cases proved that some patients were associated with a history of direct exposure to poultry or close contact with live-bird markets before disease onset, suggesting that the H7N9 virus was probably originated from poultry (Cowling et al., 2013; Liu et al., 2013b; Lu et al., 2013). During the initial survey conducted in poultry farms and live poultry markets, H7N9 viruses have been isolated mainly from bird and environmental samples collected in live bird markets, which supported the hypothesis on the source of human infections (Shi et al., 2013a). The expanding circulation of the viruses has posed a serious threat to both the public health and poultry industry of China, and the situation has even developed much worse when the highly pathogenic avian influenza (HPAI) H7N9 virus emerged in 2017 (Dong et al., 2017; Imai et al., 2017; Ke et al., 2017; Shi et al., 2017; Su et al., 2017). To eliminate the threat posed by the H7N9 virus, a compulsory vaccination in chickens has been launched by the Ministry of Agriculture and Rural Affairs of China since September 2017. This has resulted in a rapid decline of human infections, and only four human cases were reported between 2018 and 2019 (Yin et al., 2021). However, H7N9 virus has not been eradicated from poultry and the viruses have been continuously detected from poultry populations, reminding that the risk of H7N9 outbreak should not be neglected (Li and Chen, 2020).

The H7N9 virus was a reassortant of H7, N9 subtype viruses from migratory birds and avian influenza H9N2 viruses that were circulating in the poultry population of China. This virus was recognized initially as a lowly pathogenic avian influenza virus (LPAI) in chickens with a capacity of binding both human and avian cell receptors (Gao et al., 2013; Lam et al., 2013; Shi et al., 2013b; Tharakaraman et al., 2013; Xiong et al., 2013; Xu et al., 2013; Zhou et al., 2013). Several studies proved that the H7N9 virus is transmittable between ferrets through respiratory droplets, which raised great concerns about human-to-human transmission (Belser et al., 2013; Zhang et al., 2013). Although several family clusters of H7N9 infections have been reported, it is believed that the H7N9 virus hasn’t acquired the capability to transmit efficiently between humans (Liu et al., 2014; Yi et al., 2015; Wang et al., 2019; Zhang et al., 2019). However, H7N9 virus has undergone continuous evolution and reassortment with other subtypes of avian influenza viruses, and resulted in the genesis of many different genotypes of the viruses. Mutations accumulated in the genes of certain genotypes of the H7N9 virus have increased its adaptation of mammalian hosts (Li and Chen, 2020). Furthermore, 27 human cases in the fifth wave of epidemic were caused by the HPAI H7N9 viruses (Imai et al., 2017; Kang et al., 2017; Ke et al., 2017; Lee et al., 2017; Yang et al., 2017, 2018; Zhou et al., 2017; Liu et al., 2018; Yamayoshi et al., 2018), indicating that the currently circulating HPAI H7N9 viruses are more serious threats to public health.

The H7N9 virus has developed high genetic diversity and formed more than 20 genotypes (Shi et al., 2017; Li and Chen, 2020). However, the genesis and evolution of these different genotype viruses was unclear, especially in terms of spatial and temporal distribution patterns, which is the key to understand the driving forces to the evolution of a novel influenza virus. Therefore, the aim of this study was to explore the spatio-temporal spread and evolution of H7N9 virus based on their genetic connections. We conducted spatial and temporal Bayesian evolution analyzes on 2,052 H7N9 viruses, and revealed the spreading and evolutionary patterns of different genotypes of the H7N9 viruses over space and time. We also developed a random forest algorithm-based prediction model for forecasting the occurrence risks of H7N9 virus. Our findings provide new insights into the spread and evolution of H7N9 virus, and lays a theoretical basis for eliminate the potential risks for human and avian infections and provide a tool for planning risk-based virus surveillance and control.

## Materials and methods

### Data

A total of 2,052 H7N9 virus genome sequences were downloaded from the Global Initiative on Sharing All Influenza Data (GISAID)^1^ and GenBank database.^2^ The relative information on these viruses including the hosts, isolation time, and isolation sites (latitude and longitude) were compiled according to the reference materials provided during the sequence submission and the data obtained from the National Avian Influenza Reference laboratory of China.

Social and environmental factors are always associated with the occurrence of an infectious disease. To build a H7N9 occurrence risk prediction model, we also prepared a model training database, which included the data on five risk factors: the density of poultry population (DP), the density of human population (DH), the monthly average temperature (AT), the density index of water system (DIW), the number of live bird markets (NLBMs). The data on poultry productions were retrieved from the National Statistics Bureau (NSB)^3^ and the Ministry of Agriculture’s Animal Husbandry Bureau (AHB)^4^ and then standardized as DP for each county. The data on DH and AT were downloaded from the Nation Data Centre (NDC) (see text footnote 3) and the National Earth System Science Data Center,^5^ respectively. The DIW referred to the proportion of the water area (including Rivers, Lakes, and Reservoirs) to the county area. A web-based data mining program was employed to obtain the data on NLBMs in China. A H7N9 positive county/city was defined as which had at least one H7N9 virus isolated either from human, avian species, or environmental samples.

### Descriptive analysis

Previous studies showed that there were five waves of H7N9 human infections. To facilitate the comparison between human cases and viral isolation, we divided the viruses into five groups accordingly and designated Wave 1--5. An epidemic curve was drawn based on the monthly accumulated number of virus isolation. Based on the isolation numbers by the end of each wave, spatial distribution maps of H7N9 viruses against human and poultry population densities were produced by using QGIS 3.4.^6^

### Spatio-temporal Bayesian evolutionary analysis

To perform spatial-temporal Bayesian evolutionary analysis, the database was sub-divided into three subsets including HA (1,878 records), NA (1,933 recodes), and PB2 (1,827 recodes) according to the sequence quality. The HA, NA, and PB2 gene sequences were aligned using ClustalW implemented in MEGA 6.06 (Tamura et al., 2013). The spatial-temporal Bayesian evolution analysis was performed using the BEAST 1.10.0 (Drummond and Rambaut, 2007; Suchard et al., 2018) with the following settings: the HKY85 nucleotide substitution model was used to describe the process of one nucleotide being substituted for another; the uncorrelated relaxed clock with a lognormal distribution, which indicated no a priori correlation between a lineage’s rate and that of its ancestor, was applied to model rate heterogenicity; and the expansion growth with growth rate tree prior was applied for the analysis based on the occurrence dynamics of the H7N9 virus. Other parameters were set to default values as recommended by the BEAST (Shi et al., 2017). A Markov Chain Monte Carlo (MCMC) chain was selected with 10,000,000 steps and sampled every 1,000 steps. The first 10% of samples were cut-off as burn-in by the TreeAnnotator program in the BEAST package. The generated maximum clade credibility (MCC) trees were viewed in Figtree 1.4.3.^7^ Spatio-temporal evolutionary analysis based on the MCC trees with continuous traits of HA, NA, and PB2 was performed using Spread D3 (Bielejec et al., 2016).

### Genotyping of H7N9 virus

Given many genotypes of the H7N9 viruses have been developed since 2013, to explore the spatio-temporal spread and evolution of different genotypes of the H7N9 viruses, a total of 1,436 H7N9 isolates were genotyped based on the gene groups inferred by the MCC trees of HA, NA, and PB2. Then the number of the viruses belonging to each genotype was calculated at a provincial level in each wave. The temporal distribution of the genotypes was depicted by a heat map, while the spatial distribution of the genotypes was displayed on a Chinese map.

### Long-distance transmission of H7N9 virus

Based on the spatio-temporal Bayesian evolutionary analysis of the HA of H7N9 viruses, ten long-distance spreading routes were selected and analyzed. The information on these routes, including original strain, start place, stopover place, end place, start time, end time, and distance between start place and end place, were extracted and compiled. According to this information, the spreading speed of H7N9 virus for each route and an average spreading speed of the ten routes were calculated, respectively. A long-distance spread map was generated using QGIS 3.4.

### Development of occurrence risk prediction model for H7N9 virus

The prediction model was developed by using the random forest algorithm implemented in the R package *caret*. The dataset used for training and verifying the model included 2,486 records, of which 64 were positive for H7N9 virus and the rest were negatives. Seventy percent of the records were randomly selected by the algorithm and used to train the model, and the 30% rests were used to verify the model. The modeling process principally included two stages: in the first stage, the random forest algorithm generated a model through training multiple decision trees; in the second stage, the well-trained trees were employed to generate classifies. The values of the *m*_*try*_ and *ntree* were optimized to 8 and 250, respectively. We adopted a 10-fold cross-validation method to evaluate the prediction accuracy of the model. Finally, the model was employed to predict the monthly occurrence risk of H7N9 virus for each county of China. The risk maps were visualized using QGIS 3.4. The significance of the risk factors was plotted using the pheatmap tool in R 3.5.1.

## Results

### Spatial and temporal distribution of the H7N9 viruses

**FIGURE 1:**
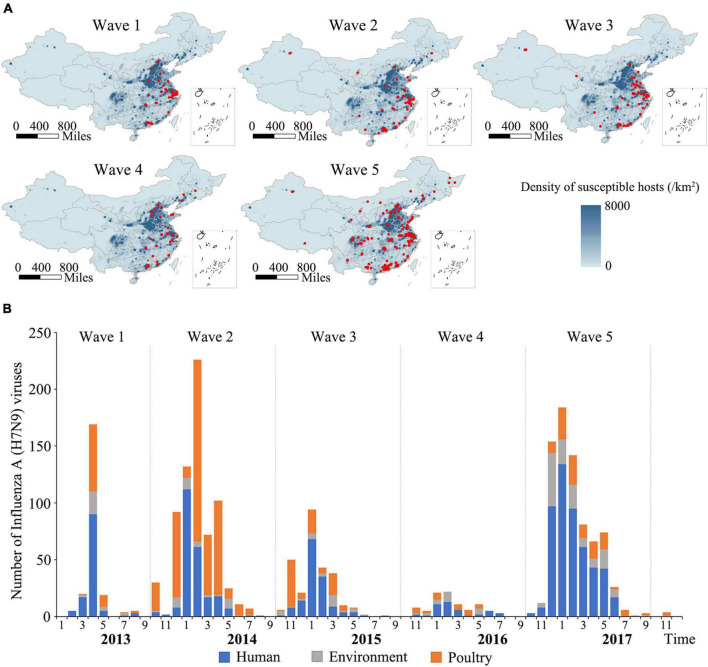
Spatial and temporal distribution of Influenza H7N9 viruses during 2013–2018. **(A)** Geographic distribution of the H7N9 viruses isolated during Wave 1–5 in mainland, China. The red circles represent locations of the viruses isolated. The background is a population density of human and poultry. The maps were generated using QGIS 3.4 (see text footnote 7). **(B)** An epidemic curve of the H7N9 viruses. The curve was generated based on the monthly cumulative numbers of the H7N9 viruses isolated during 2013–2018. Viruses isolated from human, poultry, and environment are colored blue, orange, and gray, respectively.

A total of 2,052 H7N9 viruses used in this study were classified into five groups and designated Wave 1–5 according to the time periods of previously reported human epidemics (Wang et al., 2017). The spatial distributions of the viruses are shown in Figure 1A. As indicated by the maps, the viruses isolated during Wave 1 to Wave 4 were mainly distributed in the eastern and southern regions of China, especially in Yangtze Delta Region (YDR) and Pearl Delta Region (PDR) areas, with a small number of viruses detected in other provinces, such as Hebei, Shandong, Hunan, Xinjiang, and Jilin. In contrast, a geographically striking expansion of the viruses occurred during Wave 5, with 33 out of 34 provinces or municipalities in mainland China affected, indicating an increased risk of H7N9 infections in both poultry and humans. However, the isolation of H7N9 viruses has dropped dramatically both in humans and poultry after the implementation of vaccination policy in chicken in late 2017. An epidemic curve was drawn based on monthly accumulated virus isolation number (Figure 1B). The number of the H7N9 viruses belonging to each of the waves were 222, 708, 274, 92, and 756, respectively. Only three viruses isolated after Wave 5 until 2018. These viruses were isolated from humans (50.96%), avian (36.61%), and environmental samples (12.43%). Overall, the spatio-temporal distribution patterns of the H7N9 viruses are largely consistent with that of human cases.

### Genesis and spread of the H7N9 viruses

**FIGURE 2:**
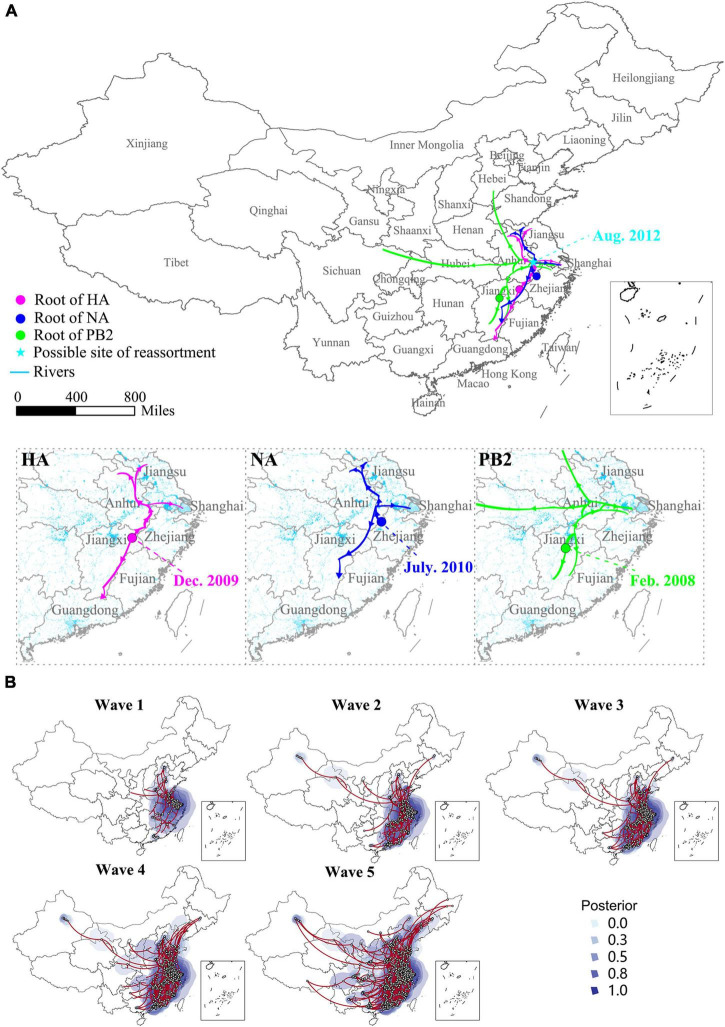
Genesis and spatio-temporal spread and evolution of the H7N9 viruses. **(A)** Genesis and initial spreading trajectory of the H7N9 virus. The large map shows the site (indicated by the cyan star) and time of the occurrence of the reassortment event. The enlarged small maps show the occurrence and initial spread route of the ancestors of the HA, NA, and PB2 of the H7N9 virus. **(B)** Spatio-temporal spread and evolution of the H7N9 viruses. The spatio-temporal Bayesian evolutionary analysis was conducted based on the gene sequences of HA of the H7N9 viruses. The analysis was performed using BEAST software with the HKY nucleotide substitution model and the expansion growth with growth rate tree prior. The maps are snapshots at the end of each wave. The posterior value shows the possibility of the occurrence of a new H7N9 virus strain.

Several studies have proved that the H7N9 virus is a multiple reassortant of avian originated influenza viruses (Chen et al., 2013; Lam et al., 2013; Liu et al., 2013a; Wu et al., 2013), but the occurrence of this reassortment event was a mystery. The spatial-temporal Bayesian evolutionary analysis based on the nucleotide sequences of HA, NA, and PB2 genes of the H7N9 viruses disclosed that the reassortment event possibly occurred in a border area of Anhui province in August 2012, which was 6 months earlier than the first human H7N9 infection reported. From then on, the viruses have spread toward the western, northern, and southern areas. Before the first human cases reported in late February 2013, the H7N9 viruses have probably presented in four provinces including Anhui, Jiangsu, Shanghai, and Jiangxi (Figure 2A). At the end of Wave 1, the majority of the H7N9 viruses circulated in YDR region, but a longitudinal spread was also found and led to the development of a new epicenter in PDR. It was noticeable that several long-distance transmissions toward northern and western provinces occurred during Wave 2, but which weren’t much developed in Wave 3 and 4. The Wave 5 was the largest among the others indicated by both the number of the isolations and their geographical distribution. An inland expanding trend was significant in this wave, resulting in almost all provinces in mainland China affected except for Hainan Province (Figure 2B and Supplementary Videos 1–3).

### Spatial and temporal evolution of the H7N9 viruses

**FIGURE 3:**
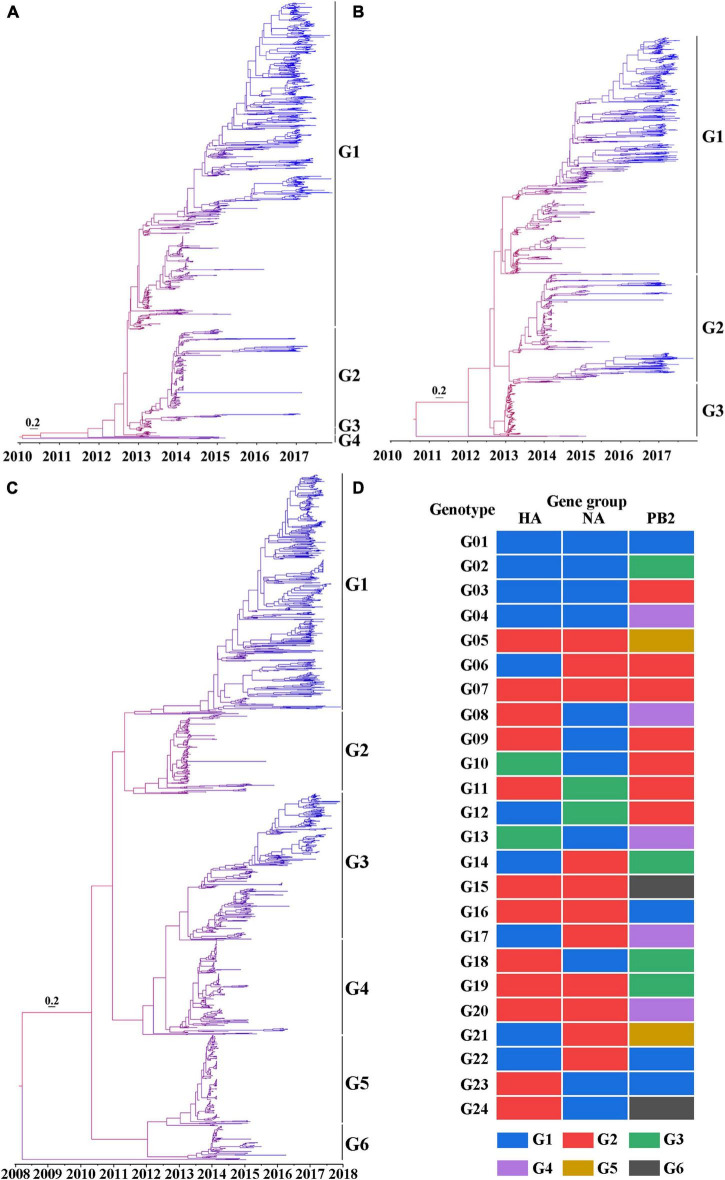
Phylogeny of the H7N9 viruses. **(A)** The maximum clade credibility (MCC) tree based on the HA nucleotide sequences of the H7N9 viruses. **(B)** The MCC tree based on the NA nucleotide sequences of the H7N9 viruses. **(C)** The MCC tree based on PB2 nucleotide sequences of the H7N9 viruses. For generating the MCC trees, the gene sequences were aligned by ClustalW in MEGA 6.06. The trees were build using BEAST 1.10.0 based on the spatio-temporal evolution analysis of gene sequences and then were visualized by Figtree 1.4.3. The color of the branches ranges from red to blue representing the tMRCA of the tree root to the most recent strains. Bar represents 0.2 calendar years. **(D)** Genotypes of the H7N9 viruses classified according to the gene lineages of HA, NA, and PB2.

To understand the evolution of the H7N9 viruses over space and time, the HA, NA, and PB2 gene sequences of 1,436 H7N9 viruses were used to develop MCC trees. According to the HA MCC tree, the H7N9 viruses were classified into four groups and designated G1-4 (Figure 3A), which is consistent with the previous reports (Shi et al., 2017). The H7N9 viruses were classified into three and six gene groups based on the MCC trees of NA (Figure 3B) and PB2 (Figure 3C), respectively. Then, those viruses were further divided into 24 genotypes according to the gene groups defined by the HA, NA, and PB2 MCC trees, and designated as genotypes G01-24 (Figure 3D).

**FIGURE 4:**
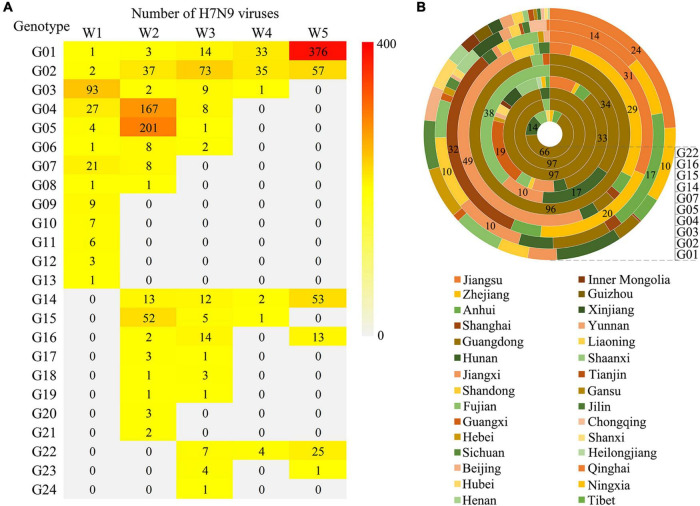
Spatio-temporal distribution of the H7N9 viruses belonging to the 24 genotypes. **(A)** The number of the viruses corresponding to each genotype isolated in each wave. **(B)** Provincial distribution of ten major genotypes. Layers of the doughnut chart from the outside to the inside represent G01, G02, G03, G04, G05, G07, G14, G15, G16, and G22, respectively. The number labels the percentage of the viruses isolated from each province.

According to their occurrence and duration, these genotypes were broadly divided into three groups: those occurred in the first wave and lasted till Wave 5, including G01 and G02; those emerged during the first wave, but disappeared before Wave 5, including G03 to G13; and those appeared after Wave 1, including G14-24. Noticeably, the G01 and G02 were the only two groups of the viruses that circulated all through the five waves with a gradual increasing in genetic diversity. A striking phenomenon was that several dominant genotypes (G03-05) in the first two waves disappeared before Wave 5, the underlying reasons need to be explored. There were also several genotypes developed after Wave 1 and became the dominant groups during Wave 5, such as G14, G16, and G22 (Figure 4A). To learn the geographical distribution of the viruses belonging to different genotypes, a doughnut chart was plotted based on the provincial isolation percentage of the top ten genotypes including G01, G02, G03, G04, G05, G07, G14, G15, G16, and G22 (Figure 4B). It revealed that these ten genotypes were grouped into two distinct spatial distribution clusters: cluster one, mainly including genotypes G01-04, was mainly isolated from the four provinces in YDR (Anhui, Jiangsu, Zhejiang, and Shanghai) and spread to other distant places, such as the northeastern and northwestern provinces; while the rest genotypes were dominantly distributed in Guangdong and Fujian provinces, with a small number of provinces affected by those genotypes of the viruses.

**FIGURE 5:**
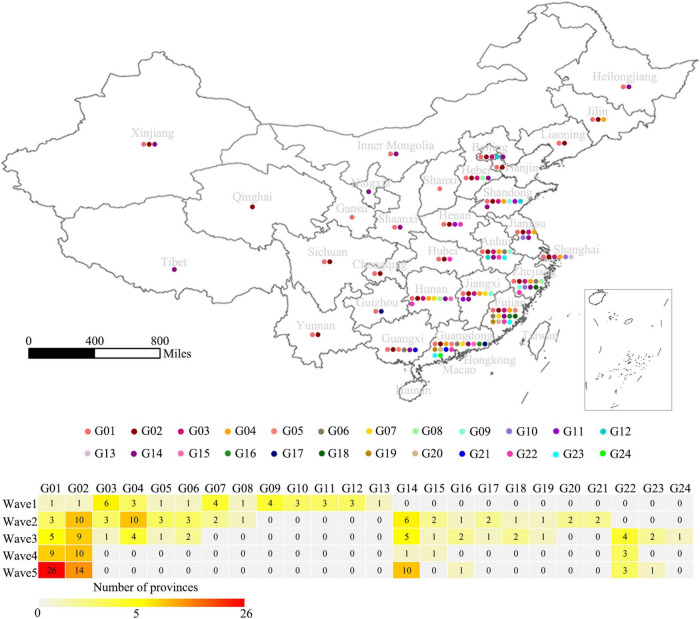
Geographical distribution of the 24 genotypes of the H7N9 viruses. The 24 genotypes are indicated by colored dots on the map of China at a provincial level. The table shows the number of affected provinces by each genotype during Wave 1–5.

To further explore the genetic diversity of the H7N9 viruses at provincial levels, a spatial distribution map of the 24 genotypes was produced and the number of the provinces affected by each of the genotypes during Wave 1–5 was calculated (Figure 5). It was very clearly that four provinces, including Guangdong, Fujian, Zhejiang, and Anhui, had the highest genetic diversities of the H7N9 viruses by showing more than ten genotypes presented and the highest of which (16 genotypes) occurred in Guangdong Province. Those provinces that having a medium number of genotypes (5 ≤ genotype number < 10) were largely the neighbors of the above four provinces. Strikingly, the number of provinces affected by the G01 and G02 groups increased steadily from Wave 1 to Wave 5, especially the G01 viruses that took over 26 provinces by the end of Wave 5. Although the viruses in G04 spread to ten provinces during Wave 2, but they disappeared after Wave 3. The G14 was another group of viruses showing a great impact in terms of geographic distribution and they emerged in Wave 2 and affected ten provinces in Wave 5.

### Long-distance transmission of H7N9 virus

**FIGURE 6:**
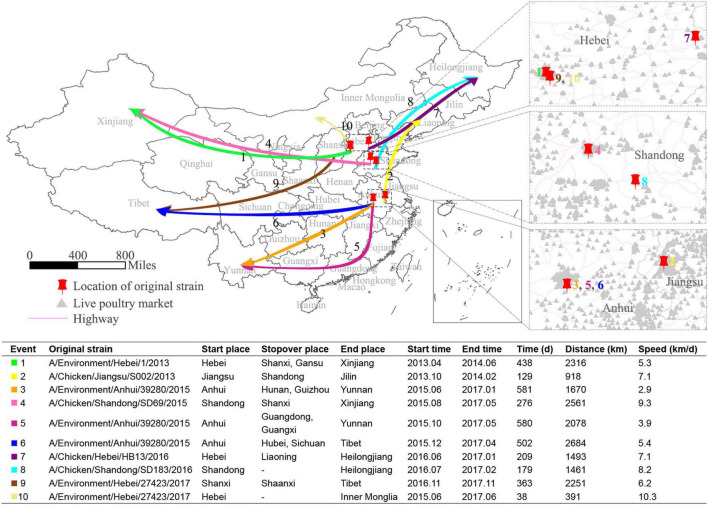
Diffusion of the H7N9 viruses over long distance. Ten long-distance spread routes of the H7N9 viruses are showed as colored arrows on the map of China. The enlarged maps display the starting places for these 10 routes, with red pushpins marking the location of the starting sites, violet lines representing highways, and light gray triangles showing the location of live bird markets. The table shows the statistics of the 10 long-distance spread events.

To understand the occurrence of long-distance spreading of the H7N9 viruses, we sampled ten of such events and the details of these events are shown in Figure 6. As expected, the sources of the viruses that led to these long-distance spreading events were environmental samples (6/10) and avian (4/10). Three of these events followed northward routes, while the rest seven followed westward routes. The distances of those transmissions varied from 391 to 2,684 km. Two of them happened between 2013 and 2014, while the others occurred during 2015–2017. About 70% of them had at least one stopover. The average speed of these long-distance spreading was 6.57 ± 2.20 km/d. The root viruses of these ten routes were almost all located in the areas with a high live bird market density, suggesting that they were transmitted probably through poultry transport systems.

### Occurrence risk of H7N9 virus

**FIGURE 7:**
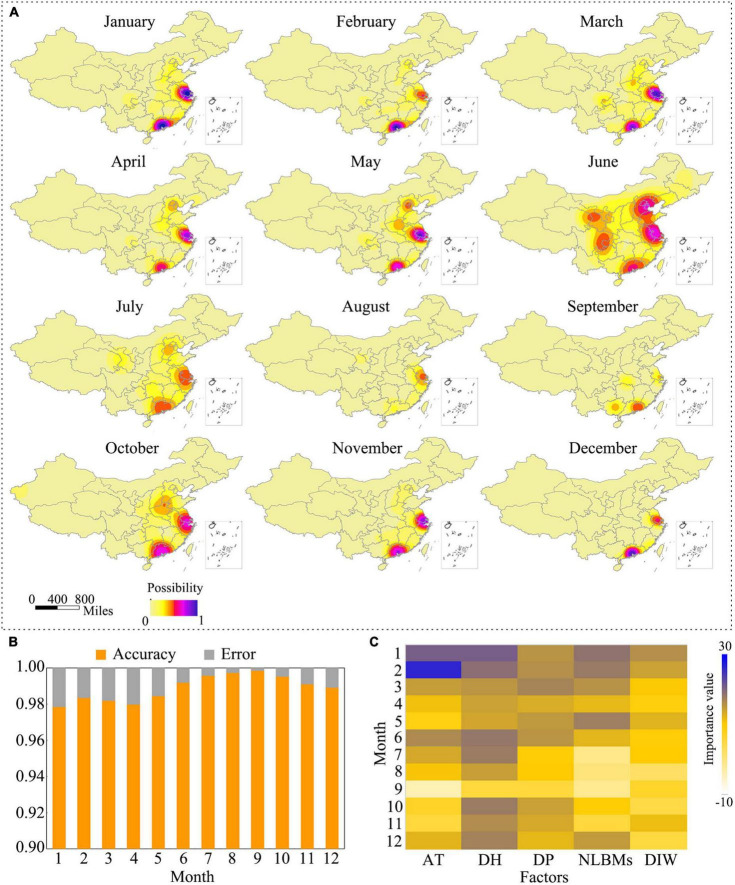
Occurrence risk of H7N9 in mainland, China. **(A)** Monthly occurrence risk of H7N9 in mainland, China. The occurrence risk was predicted using the random forest algorithm implemented in R. The risk maps were generated using QGIS 3.4. **(B)** Accuracy of the predictions. **(C)** Importance of the risk factors. “AT” represents the monthly average temperature; “DH” represents the density of human population; “DP” represents the density of poultry population; “NLBMs” represents the number of live bird markets; “DIW” represents the density index of water system.

To predict the potential occurrence risk of H7N9 virus, we developed a prediction model based on the random forest algorithm. This model involved five risk factors as we listed in the method section. The well-trained model was then used to predict the monthly occurrence risk of H7N9 virus for each county of mainland China. After kernel smoothing, the prediction results are shown in Figure 7A. It showed that the high-risk areas varied monthly with several hot spots presented in the eastern and southeastern areas of China, strikingly the YDR and PDR regions showed a constantly high risk all through the year. We also evaluated the accuracy of the prediction. It revealed the overall accuracy of the predictions was greater than 97%, and the monthly accuracy are showed in Figure 7B. The importance of each variable was assessed based on a time scale of a month. It showed that all the variables contributed differently to the monthly occurrence risk of H7N9. It seemed that all of them were important to the occurrence of H7N9 in January, February, and December, but not in other months. Comparing with the other factors, human population density was the most important one to the occurrence risk all through the year (Figure 7C).

## Discussion

In dealing with a zoonosis, integration of epidemiological evidences obtained by stakeholders, particularly the departments of public health and veterinary medicine, is the key to understand the transmission of the pathogen among human-environment-animal interface. However, it was difficult to achieve in the case of H7N9 outbreak. Initially, the H7N9 virus caused no clinical signs in poultry, making it difficult to find an infected chicken or a flock (Zhang et al., 2013). Furthermore, the rapid movements of live poultry through value chain systems hindered a retrospective investigation on the possible sources of human or poultry infections (Li et al., 2018). In this study, we connected the epidemiological data on the H7N9 viruses together by their genetic relationships, and then studied the spatio-temporal spread and evolution of the viruses, which revealed new insights into the H7N9 viruses in terms of genesis, spatial and temporal distributions, major spreading routes, and potential risks of human and avian infections.

Previous studies have revealed that the original H7N9 virus is a reassortant of wild bird, duck, and chicken source viruses (Gao et al., 2013; Lam et al., 2013; Liu et al., 2013a). But the occurrence of the reassortment event was unclear. Therefore, we depicted this process based on the Bayesian evolutionary analysis. Given that the HA, NA, and the internal genes of the H7N9 virus were obtained from different origins, we therefore conducted Bayesian evolutionary analyzes based on the gene sequences of HA, NA, and PB2 (representing the internal genes) of the H7N9 viruses, respectively. These analyzes revealed that the root viruses bearing the HA, NA, and PB2 of the H7N9 virus emerged in different locations and times. The reassortment event happened probably in an area in Anhui Province when these three types of the viruses met each other in August 2012. This place is located by the Yangtze River and near the Tai Lake, and is one of the major habituating places for many migratory bird species in winter. The local areas have a high density of chicken and waterfowl populations. The natural and social environment provided a suitable condition for the genesis of a reassortant influenza virus. The predicted reassortment event occurred about 6 months earlier than the first human case reported in late February in 2013. The modeling disclosed that the virus had already presented in four provinces including Anhui, Jiangsu, Shanghai, and Jiangxi before the time of human infections, which was supported by a survey conducted in poultry populations that showed all these provinces were positive for the H7N9 virus isolation (Zhang et al., 2013).

Avian influenza viruses, especially the HPAIV, are always characterized by rapid spreading among poultry populations. However, the spread of the H7N9 virus was relatively slower. During the initial wave of the epidemics, most of the viruses clustered in the YDR epicenter and circulated locally, only with a small number of the virus spread to two farthest destinations of Beijing in the north and Guangdong in the south. The later site has developed into another epicenter designated PDR. The low spreading speed of the H7N9 virus was possibly contributed by their low pathogenicity in chickens and less adaptation to mammalian hosts (Zaraket et al., 2015). With the accumulation of the mutations in the viral proteins, their capability to adapt to mammalian and avian hosts has increased continuously, which could explain in partially the observation of a rapidly geographical expansion occurred during Wave 5. The common features of the two epicenters include high densities of chickens, waterfowls, water system, live-bird markets, and human population as well, suggesting that areas having similar conditions with these two epicenters might be suitable circumstances for an avian influenza virus to root and spread.

The H7N9 virus has been undergoing continuous evolution and reassortment, resulting in increased biological characteristics and genetic diversity. The evolution of the H7N9 virus has been intensively investigated. It showed that at least 23 genetic lineages and mutations had been developed in the H7N9 viruses by the end of 2017 (Shi et al., 2017). In this study, we investigated the evolution of 1,436 H7N9 viruses based on the MCC trees of HA, NA, and PB2. These viruses were placed into 24 genotypes (G01∼24), which was largely consistent with the above-mentioned results. In addition, our study revealed in details about the evolving process, geographical distribution, and final fate of each genotype viruses. The distinct spatial and temporal patterns of the genotypes of the H7N9 viruses are valuable for generating hypotheses about the risk factors that associated with the spread of the viruses, and identifying the key amino acid residues in the viral proteins that affected the transmission and pathogenicity of the H7N9 viruses in different host species. Interestingly, although the H7N9 viruses emerged in YDR, the highest genetic diversity of the viruses has been developed in PDR, from where 16 out of the 24 genotypes of the viruses were isolated, but the affected areas by these groups of the viruses were smaller than by the G01 viruses. One of the reasons was that the poultry outward transportation in YDR was higher than that in PDR, and the true mechanism need to be explored in the future. It’s worth noting that several genotypes, such as G08 to G13, disappeared in a relatively short period of time for unknown reasons. One possible explanation is that they died out because either of the control measures taken in the infected chicken population or they did not adapt well in their host species.

It was believed that the movement of live poultry has led to the spread of H7N9 virus (Zhou et al., 2015). According to the poultry transportation system of China, an infected flock can be transported to most areas of China within 2 days (Fang et al., 2008; Yang et al., 2020). To estimate the spread speed of the H7N9 virus, we sampled ten long-distance spreading events and calculated the spreading speed of the viruses. It showed that the average spreading speed of the H7N9 viruses was 6.57 ± 2.20 km/d, which means that almost 10 days were needed for a H7N9 virus to spillover from an infected county to its neighboring counties in China. This created a favorite condition for controlling H7N9 in poultry population through a vaccination campaign. Furthermore, the root viruses leading to the long-distance transmissions were all originated from chicken or environmental samples, suggesting that eradicating the viruses in poultry populations was the key to control the spread and human infections with the H7N9 virus.

Given the concurrence of H7N9 virus showed obvious spatial and temporal patterns, combining with the previous studies showing that many social and environmental risk factors were associated with the outbreak of H7N9 (Zhou et al., 2015; Artois et al., 2018; Zheng et al., 2020), we, therefore, determined to build a model to predict the occurrence risk of H7N9. Machine learning methods are valuable in many fields, especially with the boom of big data acquisition and treating techniques, its applications have shown a spurt growth (Ngiam and Khor, 2019; Goodswen et al., 2021). In the field of infectious diseases, many predictive models based on the machine learning methods have been developed and successfully applied in predicting the disease outbreak risks (Gussow et al., 2020; Carlson et al., 2021). We then built an occurrence prediction model for H7N9 virus based on the random forest algorithms, which is one of the most popular machine learning algorithms in the field of artificial intelligence. A total of 2,486 records were prepared as the training database, and 70% of them were used to train the model, the rest were used to test its accuracy. The assessment showed an overall 97% accuracy of the model, suggesting that this model was in a good performance in predicting H7N9 virus. The model also revealed the importance of each indicator. Risk-based surveillance on animal diseases, especially in developing countries, has been advocated by FAO (The Food and Agriculture) and OIE (the International Organization of Animal Health) recently. The predicted H7N9 risks provided monthly based geographical targets for conducting risk-based H7N9 virus surveillance and eliminating the potential outbreak risks. Furthermore, this model can be adapted to predict the risks for other zoonoses.

In summary, influenza A (H7N9) virus has developed high genetic diversity and affected more than 30 provinces in mainland China during 2013–2018. This study has profiled the process in detail and identified the spatio-temporal distribution patterns of each genotype H7N9 viruses, which are valuable for exploring the environmental and genetic factors associated with those patterns and eliminating potential disease risks in the future.

## Data availability statement

The data used for conducting the spatial and temporal Bayesian evolutionary analysis are not shared publicly. Individuals who want to access data used in this study should contact the corresponding author (wangjingfei@caas.cn) for permission.

## Author contributions

JW and YJ conceived the study. ZS, LW, PW, SW, and ZL collected the data. ZS, LW, YJ, and JW analyzed data. ZS, YJ, and JW wrote the manuscript. All authors discussed and commented on the results and the manuscript.

## Footnotes

1
http://www.gisaid.org

2
http://www.ncbi.nlm.nih.gov

3
https://data.stats.gov.cn/english/

4
http://english.moa.gov.cn/

5
http://gre.geodata.cn

6
https://www.qgis.org

7
http://tree.bio.ed.ac.uk/software/figtree/

## Supplementary material

Supplementary Video 1 Spatio-temporal evolution of H7N9 virus referred to the analysis of HA gene. A total of 1,878 HA sequences were aligned using ClustalW implemented in MEGA, and then used to perform the spatial-temporal Bayesian evolution analysis by BEAST. Spatial-temporal evolutionary analysis based on the generated maximum clade credibility (MCC) tree with continuous traits of HA was performed using SpreadD3.

Supplementary Video 2 Spatio-temporal evolution of H7N9 virus referred to the analysis of NA gene. A total of 1,933 NA sequences were aligned using ClustalW implemented in MEGA, and then used to perform the spatial-temporal Bayesian evolution analysis by BEAST with parameters that mentioned in method section. Spatial-temporal evolutionary analysis based on the generated maximum clade credibility (MCC) tree with continuous traits of NA was performed using SpreadD3.

Supplementary Video 3 Spatio-temporal evolution of H7N9 virus referred to the analysis of PB2 gene. A total of 1,827 PB2 sequences were aligned using ClustalW implemented in MEGA, and then used to perform the spatial-temporal Bayesian evolution analysis by BEAST with parameters that mentioned in method section. Spatial-temporal evolutionary analysis based on the generated maximum clade credibility (MCC) tree with continuous traits of PB2 was performed using SpreadD3.

## References

[B1] Artois J. Jiang H. Wang X. Qin Y. Pearcy M. Lai S. (2018). Changing geographic patterns and risk factors for avian influenza A(H7N9) infections in humans, China. *Emerg. Infect. Dis.* 24 87–94. 10.3201/eid2401.171393 29260681 PMC5749478

[B2] Belser J. A. Gustin K. M. Pearce M. B. Maines T. R. Zeng H. Pappas C. (2013). Pathogenesis and transmission of avian influenza A (H7N9) virus in ferrets and mice. *Nature* 501 556–559. 10.1038/nature12391 23842497 PMC7094885

[B3] Bielejec F. Baele G. Vrancken B. Suchard M. A. Rambaut A. Lemey P. (2016). SpreaD3: interactive visualization of spatiotemporal history and trait evolutionary processes. *Mol. Biol. Evol.* 33 2167–2169. 10.1093/molbev/msw082 27189542 PMC6398721

[B4] Carlson C. J. Farrell M. J. Grange Z. Han B. A. Mollentze N. Phelan A. L. (2021). The future of zoonotic risk prediction. *Philos. Trans. R. Soc. Lond. B Biol. Sci.* 376:20200358. 10.1098/rstb.2020.0358 34538140 PMC8450624

[B5] Chen Y. Liang W. Yang S. Wu N. Gao H. Sheng J. (2013). Human infections with the emerging avian influenza A H7N9 virus from wet market poultry: clinical analysis and characterisation of viral genome. *Lancet* 381 1916–1925. 10.1016/S0140-6736(13)60903-4 23623390 PMC7134567

[B6] Cowling B. J. Jin L. Lau E. H. Liao Q. Wu P. Jiang H. (2013). Comparative epidemiology of human infections with avian influenza A H7N9 and H5N1 viruses in China: a population-based study of laboratory-confirmed cases. *Lancet* 382 129–137. 10.1016/S0140-6736(13)61171-X 23803488 PMC3777567

[B7] Dong W. Yang K. Xu Q. Liu L. Chen J. (2017). Spatio-temporal pattern analysis for evaluation of the spread of human infections with avian influenza A(H7N9) virus in China, 2013-2014. *BMC Infect. Dis.* 17:704. 10.1186/s12879-017-2781-2782 PMC5655814 29065855

[B8] Drummond A. J. Rambaut A. (2007). BEAST: Bayesian evolutionary analysis by sampling trees. *BMC Evol. Biol.* 7:214. 10.1186/1471-2148-7-214 17996036 PMC2247476

[B9] Fang L. Q. de Vlas S. J. Liang S. Looman C. W. Gong P. Xu B. (2008). Environmental factors contributing to the spread of H5N1 avian influenza in mainland China. *PLoS One* 3:e2268. 10.1371/journal.pone.0002268 18509468 PMC2386237

[B10] Gao R. Cao B. Hu Y. Feng Z. Wang D. Hu W. (2013). Human infection with a novel avian-origin influenza A (H7N9) virus. *N. Engl. J. Med.* 368 1888–1897. 10.1056/NEJMoa1304459 23577628

[B11] Goodswen S. J. Barratt J. L. N. Kennedy P. J. Kaufer A. Calarco L. Ellis J. T. (2021). Machine learning and applications in microbiology. *FEMS Microbiol. Rev.* 45:fuab015. 10.1093/femsre/fuab015 33724378 PMC8498514

[B12] Gussow A. B. Auslander N. Wolf Y. I. Koonin E. V. (2020). Prediction of the incubation period for COVID-19 and future virus disease outbreaks. *BMC Biol.* 18:186. 10.1186/s12915-020-00919-919 PMC7703724 33256718

[B13] Imai M. Watanabe T. Kiso M. Nakajima N. Yamayoshi S. Iwatsuki-Horimoto K. (2017). A highly pathogenic avian H7N9 influenza virus isolated from a human is lethal in some ferrets infected via respiratory droplets. *Cell Host Microbe* 22 615–626.e8. 10.1016/j.chom.2017.09.008. 29056430 PMC5721358

[B14] Kang M. Lau E. H. Y. Guan W. Yang Y. Song T. Cowling B. J. (2017). Epidemiology of human infections with highly pathogenic avian influenza A(H7N9) virus in Guangdong, 2016 to 2017. *Euro. Surveill.* 22:30568. 10.2807/1560-7917.ES.2017.22.27.30568 PMC5508330 28703705

[B15] Ke C. Mok C. K. P. Zhu W. Zhou H. He J. Guan W. (2017). Human infection with highly pathogenic avian influenza A(H7N9) virus. *China. Emerg. Infect. Dis.* 23 1332–1340. 10.3201/eid2308.170600 28580899 PMC5547808

[B16] Lam T. T. Wang J. Shen Y. Zhou B. Duan L. Cheung C. L. (2013). The genesis and source of the H7N9 influenza viruses causing human infections in China. *Nature* 502 241–244. 10.1038/nature12515 23965623 PMC3801098

[B17] Lee D. H. Torchetti M. K. Killian M. L. Berhane Y. Swayne D. E. (2017). Highly pathogenic avian influenza A(H7N9) virus, tennessee, USA, March 2017. *Emerg. Infect. Dis.* 23 1860–1863. 10.3201/eid2311.171013 28880836 PMC5652434

[B18] Li C. Chen H. (2020). H7N9 influenza virus in China. *Cold Spring Harb Perspect Med.* 11:a038349. 10.1101/cshperspect.a038349 32205415 PMC8327827

[B19] Li Y. Wang Y. Shen C. Huang J. Kang J. Huang B. (2018). Closure of live bird markets leads to the spread of H7N9 influenza in China. *PLoS One* 13:e0208884. 10.1371/journal.pone.0208884 30540847 PMC6291110

[B20] Liu D. Shi W. Shi Y. Wang D. Xiao H. Li W. (2013a). Origin and diversity of novel avian influenza A H7N9 viruses causing human infection: phylogenetic, structural, and coalescent analyses. *Lancet* 381 1926–1932. 10.1016/S0140-6736(13)60938-1 23643111

[B21] Liu W. Zhu Y. Qi X. Xu K. Ge A. Ji H. (2013b). Risk assessment on the epidemics of human infection with a novel avian influenza A (H7N9) virus in Jiangsu Province, China. *J. Biomed. Res.* 27 163–166. 10.7555/JBR.27.20130071 23720670 PMC3664721

[B22] Liu T. Bi Z. Wang X. Li Z. Ding S. Bi Z. (2014). One family cluster of avian influenza A(H7N9) virus infection in Shandong, China. *BMC Infect. Dis.* 14:98. 10.1186/1471-2334-14-98 24559386 PMC3974040

[B23] Liu D. Zhang Z. He L. Gao Z. Li J. Gu M. (2018). Characteristics of the emerging chicken-origin highly pathogenic H7N9 viruses: a new threat to public health and poultry industry. *J. Infect.* 76 217–220. 10.1016/j.jinf.2017.09.005 28941628

[B24] Lu S. Zheng Y. Li T. Hu Y. Liu X. Xi X. (2013). Clinical findings for early human cases of influenza A(H7N9) virus infection, Shanghai, China. *Emerg. Infect. Dis.* 19 1142–1146. 10.3201/eid.1907.130612 23769184 PMC3713996

[B25] Ngiam K. Y. Khor I. W. (2019). Big data and machine learning algorithms for health-care delivery. *Lancet Oncol.* 20 e262–e273. 31044724 10.1016/S1470-2045(19)30149-4

[B26] Shi J. Deng G. Kong H. Gu C. Ma S. Yin X. (2017). H7N9 virulent mutants detected in chickens in China pose an increased threat to humans. *Cell Res.* 27 1409–1421. 10.1038/cr.2017.129 29151586 PMC5717404

[B27] Shi J. Deng G. Liu P. Zhou J. Guan L. Li W. (2013a). Isolation and characterization of H7N9 viruses from live poultry markets — implication of the source of current H7N9 infection in humans. *Chinese Sci. Bull.* 58 1857–1863. 10.1007/s11434-013-5873-5874

[B28] Shi Y. Zhang W. Wang F. Qi J. Wu Y. Song H. (2013b). Structures and receptor binding of hemagglutinins from human-infecting H7N9 influenza viruses. *Science* 342 243–247. 10.1126/science.1242917 24009358

[B29] Su S. Gu M. Liu D. Cui J. Gao G. F. Zhou J. (2017). Epidemiology, evolution, and pathogenesis of H7N9 influenza viruses in five epidemic waves since 2013 in China. *Trends Microbiol.* 25 713–728. 10.1016/j.tim.2017.06.008 28734617

[B30] Suchard M. A. Lemey P. Baele G. Ayres D. L. Drummond A. J. Rambaut A. (2018). Bayesian phylogenetic and phylodynamic data integration using BEAST 1.10. *Virus Evol.* 4:vey016. 10.1093/ve/vey016 29942656 PMC6007674

[B31] Tamura K. Stecher G. Peterson D. Filipski A. Kumar S. (2013). MEGA6: molecular evolutionary genetics analysis version 6.0. *Mol. Biol. Evol.* 30 2725–2729. 10.1093/molbev/mst197 24132122 PMC3840312

[B32] Tharakaraman K. Jayaraman A. Raman R. Viswanathan K. Stebbins N. W. Johnson D. (2013). Glycan receptor binding of the influenza a virus H7N9 hemagglutinin. *Cell* 153 1486–1493. 10.1016/j.cell.2013.05.034 23746830 PMC3746546

[B33] Wang X. Jiang H. Wu P. Uyeki T. M. Feng L. Lai S. (2017). Epidemiology of avian influenza A H7N9 virus in human beings across five epidemics in mainland China, 2013-17: an epidemiological study of laboratory-confirmed case series. *Lancet Infect. Dis.* 17 822–832. 10.1016/S1473-3099(17)30323-7 28583578 PMC5988584

[B34] Wang X. Wu P. Pei Y. Tsang T. K. Gu D. Wang W. (2019). Assessment of human-to-human transmissibility of avian influenza A(H7N9) virus across 5 waves by analyzing clusters of case patients in mainland China, 2013-2017. *Clin. Infect. Dis.* 68 623–631. 10.1093/cid/ciy541 29961834 PMC6355824

[B35] Wu A. Su C. Wang D. Peng Y. Liu M. Hua S. (2013). Sequential reassortments underlie diverse influenza H7N9 genotypes in China. *Cell Host Microbe* 14 446–452. 10.1016/j.chom.2013.09.001 24055604

[B36] Xiang D. Pu Z. Luo T. Guo F. Li X. Shen X. (2018). Evolutionary dynamics of avian influenza a H7N9 virus across five waves in mainland China, 2013-2017. *J. Infect.* 77 205–211. 10.1016/j.jinf.2018.05.006 29807090

[B37] Xiong X. Martin S. R. Haire L. F. Wharton S. A. Daniels R. S. Bennett M. S. (2013). Receptor binding by an H7N9 influenza virus from humans. *Nature* 499 496–499. 10.1038/nature12372 23787694

[B38] Xu R. de Vries R. P. Zhu X. Nycholat C. M. McBride R. Yu W. (2013). Preferential recognition of avian-like receptors in human influenza a H7N9 viruses. *Science* 342 1230–1235. 10.1126/science.1243761 24311689 PMC3954636

[B39] Yamayoshi S. Kiso M. Yasuhara A. Ito M. Shu Y. Kawaoka Y. (2018). Enhanced replication of highly pathogenic influenza A(H7N9) virus in humans. *Emerg. Infect. Dis.* 24 746–750. 10.3201/eid2404.171509 29553313 PMC5875272

[B40] Yang L. Zhu W. Li X. Chen M. Wu J. Yu P. (2017). Genesis and spread of newly emerged highly pathogenic H7N9 avian viruses in mainland China. *J. Virol.* 91:e01277-17. 10.1128/JVI.01277-1217 PMC5686710 28956760

[B41] Yang Q. Shi W. Zhang L. Xu Y. Xu J. Li S. (2018). Westward spread of highly pathogenic avian influenza a(H7N9) virus among humans, China. *Emerg. Infect. Dis.* 24 1095–1098. 10.3201/eid2406.171135 29619922 PMC6004833

[B42] Yang Q. Zhao X. Lemey P. Suchard M. A. Bi Y. Shi W. (2020). Assessing the role of live poultry trade in community-structured transmission of avian influenza in China. *Proc. Natl. Acad. Sci. U S A.* 117 5949–5954. 10.1073/pnas.1906954117 32123088 PMC7084072

[B43] Yi L. Guan D. Kang M. Wu J. Zeng X. Lu J. (2015). Family clusters of avian influenza a H7N9 virus infection in Guangdong Province, China. *J. Clin. Microbiol.* 53 22–28. 10.1128/JCM.02322-2314 25339399 PMC4290927

[B44] Yin X. Deng G. Zeng X. Cui P. Hou Y. Liu Y. (2021). Genetic and biological properties of H7N9 avian influenza viruses detected after application of the H7N9 poultry vaccine in China. *PLoS Pathog* 17:e1009561. 10.1371/journal.ppat.1009561 33905456 PMC8104392

[B45] Zaraket H. Baranovich T. Kaplan B. S. Carter R. Song M. S. Paulson J. C. (2015). Mammalian adaptation of influenza A(H7N9) virus is limited by a narrow genetic bottleneck. *Nat. Commun.* 6:6553. 10.1038/ncomms7553 25850788 PMC4403340

[B46] Zhang Q. Shi J. Deng G. Guo J. Zeng X. He X. (2013). H7N9 influenza viruses are transmissible in ferrets by respiratory droplet. *Science* 341 410–414. 10.1126/science.1240532 23868922

[B47] Zhang W. Zhao K. Jin J. He J. Zhou W. Wu J. (2019). A hospital cluster combined with a family cluster of avian influenza H7N9 infection in Anhui Province, China. *J. Infect.* 79 49–55. 10.1016/j.jinf.2019.05.008 31100362 PMC7112695

[B48] Zheng S. Zou Q. Wang X. Bao J. Yu F. Guo F. (2020). Factors associated with fatality due to avian influenza A(H7N9) infection in China. *Clin. Infect. Dis.* 71 128–132. 10.1093/cid/ciz779 31418813 PMC8127055

[B49] Zhou J. Wang D. Gao R. Zhao B. Song J. Qi X. (2013). Biological features of novel avian influenza A (H7N9) virus. *Nature* 499 500–503. 10.1038/nature12379 23823727

[B50] Zhou L. Tan Y. Kang M. Liu F. Ren R. Wang Y. (2017). Preliminary epidemiology of human infections with highly pathogenic avian influenza A(H7N9) virus, China, 2017. *Emerg. Infect. Dis.* 23 1355–1359. 10.3201/eid2308.170640 28580900 PMC5547798

[B51] Zhou X. Li Y. Wang Y. Edwards J. Guo F. Clements A. C. (2015). The role of live poultry movement and live bird market biosecurity in the epidemiology of influenza A (H7N9): a cross-sectional observational study in four eastern China provinces. *J. Infect.* 71 470–479. 10.1016/j.jinf.2015.06.012 26149187

[B52] Zhu W. Dong J. Zhang Y. Yang L. Li X. Chen T. (2018). A gene constellation in avian influenza A (H7N9) viruses may have facilitated the fifth wave outbreak in China. *Cell Rep.* 23 909–917. 10.1016/j.celrep.2018.03.081 29669294

